# INSTRUMENTAL ACTIVITIES OF DAILY LIVING (IADLS) PREDICT PLASMA NFL LEVELS IN OLDER ADULTS WITHOUT DEMENTIA

**DOI:** 10.1093/geroni/igae098.1012

**Published:** 2024-12-31

**Authors:** Mengchi Li, Russell Calderon, Sofia Liu, Junxin Li

**Affiliations:** Xi’an Jiaotong University, Xi’an, Shaanxi, China (People’s Republic); Johns Hopkins University, Lehigh Acres, Florida, United States; Johns Hopkins University School of Nursing, Baltimore, Maryland, United States; Johns Hopkins School of Nursing, Baltimore, Maryland, United States

## Abstract

Instrumental Activities of Daily Living (IADLs) are crucial for the independent living of older adults. Subtle declines in IADLs have been associated with decreased cognitive function. Yet, the knowledge regarding the association of IADL and plasma Neurofilament light chain (NfL), a biomarker for neurodegeneration, remains limited. This study aimed to examine the cross-sectional association between IADL and NfL in 102 older adults without dementia (Age≥65 years, Montreal Cognitive Assessment (MoCA)> 17), using baseline data collected from a randomized clinical trial (NCT03959202). The sample was 70.3 ± 6.1 years old, with 79% being females, 61% being White American, 33% being Black or African American, 82% having intact cognition, and 18% having mild cognitive impairment. IADLs were assessed using the Lawton Instrumental Activities of Daily Living Scale. Plasma NfL levels were examined continuously and dichotomously (≥ 75% vs. < 75%) as the study outcome. Multiple linear and logistic regression analyses were conducted with IADLs total score as the primary predictor and adjusting for age, sex, education, chronic conditions, and MoCA. Higher IADL (better function) was associated with lower plasma NfL level (β=-4.02, 95% confidence interval [CI]: -5.99, -2.06). The logistic regression model showed that 1 point increase in IADLs score was associated with 67% reduced odds of having high NfL (OR: 0.33, 95% CI: 0.14, 0.77). In conclusion, we found that higher instrumental activities of daily living were associated with decreased plasma NfL, suggesting a mechanism underlying functional decline and neurodegeneration in non-demented older adults.

